# 2681. National Outpatient Sexually Transmitted Infection Testing in Pregnant Patients in the United States, 2012 to 2019

**DOI:** 10.1093/ofid/ofad500.2292

**Published:** 2023-11-27

**Authors:** Kelsey Strey, Vidal M Mendoza, Kelly Reveles

**Affiliations:** The University of Texas at Austin College of Pharmacy, San Antonio, Texas; UT College of Pharmacy, San Antonio, Texas; The University of Texas at Austin, San Antonio, Texas

## Abstract

**Background:**

Sexually transmitted infections (STI) continue to increase annually in the United States (US) with 2.5 million infections reported by the Centers for Disease Control and Prevention (CDC) in 2021. Pregnant patients are at risk for serious complications due to infection. It is recommended that pregnant women are screened early in pregnancy to avoid poor outcomes; however, few studies have used nationally representative data to assess STI testing in pregnancy in the US. This study aimed to estimate the utilization of preventative STI care on a national scale in pregnant patients.

**Methods:**

This was a retrospective, cross-sectional study of outpatient visits in the CDC's National Ambulatory Medical Care Survey from 2012 to 2019. All patients reported as pregnant were included for analysis to assess STI testing (e.g., chlamydia, gonorrhea, hepatitis, HIV) overall. STI testing was described per 1000 total visits. Subgroup analyses were conducted by race, ethnicity and region. Multivariable logistic regression was used to identify predictors of receiving at least one STI test. Data weights were applied to analyses to generate national estimates.

**Results:**

Over 232 million visits were included for analysis, of which 7.6% included an STI test. Overall, rates of receiving any STI test varied over the study period (39.6-136.0 per 1000 visits). Chlamydia testing was the most common, followed by gonorrhea, HIV, and hepatitis (48.5 v. 42.1 v. 39.5 v. 19.6 per 1000). Patients of non-Hispanic ethnicity, White race, and those in the West region had the lowest rates of STI testing (74.1 v. 55.8 v. 43.4 per 1000). Overall, Hispanic ethnicity (aOR 1.152, 95%CI 1.150-1.155), Black and more than one race (aOR 2.837, 95%CI 2.831-2.843 and aOR 3.06, 95%CI 3.04-3.09), government and private insurance (aOR 4.63, 95%CI 4.58-4.68 and aOR 4.71, 95%CI 4.66-4.76), metropolitan areas (aOR 1.865, 95%CI 1.859-1.871), and the Northeast region (aOR 2.449, 95%CI 2.442-2.455) were predictive of receiving any STI test.

**Conclusion:**

STI testing in US outpatient physician offices varied over the study period and across test types, though was consistently low. Additionally, certain patient attributes, such as race, ethnicity, and payment source were predictive of testing.

**Disclosures:**

**Kelly Reveles, PharmD, PhD**, Ferring Pharmaceuticals: Advisor/Consultant|Ferring Pharmaceuticals: Honoraria

